# Polydrug abuse among opioid maintenance treatment patients is related to inadequate dose of maintenance treatment medicine

**DOI:** 10.1186/s12888-017-1415-y

**Published:** 2017-07-06

**Authors:** Pertti Kalevi Heikman, Leea Hellevi Muhonen, Ilkka Antero Ojanperä

**Affiliations:** 1 University of Helsinki and Helsinki University Central Hospital, Psychiatry, P.O. Box 22, Välskärinkatu 12 A, FI-00014 Helsinki, Finland; 2 University of Helsinki, Forensic Medicine, P.O. Box 40, Kytösuontie 11, FI-00014 Helsinki, Finland; 3 National Institute for Health and Welfare, Forensic Toxicology Unit, P.O. Box 30, Mannerheimintie 166, FI-00271 Helsinki, Finland

**Keywords:** Opioid maintenance treatment, Polydrug abuse, Dose, Drug screening, Time-of-flight mass spectrometry

## Abstract

**Background:**

Polydrug abuse is a known problem among opioid-dependent patients receiving opioid maintenance treatment (OMT). However, improved laboratory diagnostics is required to reveal polydrug abuse in its current scope. Furthermore, there are few studies focusing on the relationship between polydrug abuse and adequacy of the dose of OMT medicine. This study aimed to evaluate the polydrug abuse among opioid-dependent patients receiving OMT with inadequate (Group IA) and adequate (Group A) doses of OMT medicine as experienced by the patients. Craving for opioids and withdrawal symptoms were evaluated as indicators of the adequacy rating.

**Methods:**

This is a retrospective register-based study of 60 OMT patients on either methadone or sublingual buprenorphine/naloxone medication, whose polydrug abuse was studied from urine samples by means of a comprehensive high-resolution mass spectrometry method.

**Results:**

Inadequate doses of the OMT medicines were associated with higher subjective withdrawal scores and craving for opioids. Six groups of abused substances (benzodiazepines, amphetamines, opioids, cannabis, new psychoactive substances, and non-prescribed psychotropic medicines) were found among OMT patients. Group IA patients showed significantly more abuse of benzodiazepines and amphetamines than the Group A patients. All the new psychoactive substances and most of the non-prescribed psychotropic medicines were detected from the Group IA patients. There was no difference in the doses of the OMT medicine between Groups IA and A patients.

**Conclusions:**

Polydrug abuse, detected by definitive laboratory methods, was widespread and more common among Group IA than Group A patients, emphasizing the requirement for individual OMT medicine dose adjustment.

## Background

Maintenance treatment for opioid dependence (OMT), either with methadone or buprenorphine-based medications, improves retention in the treatment and reduces abuse of illicit opioids [1]. Many OMT studies have targeted patients with heroin dependence and cocaine abuse [2, 3]. However, in Finland the most commonly abused opioid is buprenorphine, and the most commonly abused stimulant is amphetamine [4]. Consequently, the results concerning heroin-dependent patients may not be applicable to the Finnish OMT patients. Yet, OMT does not prevent polydrug abuse. Polydrug abuse during OMT is a significant problem based on surveys [4, 5] and on studies relying on standard immunoassay screening [6]. Broad-spectrum polydrug abuse includes not only traditional illicit drugs, but also non-prescribed psychotropic medicines (N-PPM) and new psychoactive substances (NPS) [1, 7]. For the first time, the data concerning NPS reports for 2015 [7] discloses a wide range of substances (e.g. fentanyl derivates and benzodiazepines) not belonging to any of the major groups identified in previous years. The abuse of BZD among OMT patients is worldwide, with a reported prevalence of 45% in France and 70% in Germany [8], 51% in Israel [9], 47% in the USA [10], and 38% in Australia [11]. Polydrug abuse for its part decreases retention in OMT [12], and the decreased retention is related to the degree of severity of polydrug abuse [13].

Most NPS and many N-PPM are not detectable by conventional urine immunoassays, but a broad range of these new abused drugs, in addition to traditional illicit drugs, can be revealed by comprehensive urine drug-screening methods based especially on liquid chromatography-high-resolution time-of-flight mass spectrometry (LC-TOFMS) [14]. Using this type of urine analysis, polydrug abuse, including NPS and N-PPM, has also been reported among OMT patients [15–18]. The LC-TOFMS method has proved to be specific and sensitive, providing a scope and reliability beyond standard immunoassay, without the necessity for a subsequent quantitative confirmation analysis [14].

Both methadone and buprenorphine in fixed doses are effective in suppressing illicit opioid use [2, 19–21]. Fixed-dose studies do not require individualized dose levels because of retention in treatment, and treatment outcomes. However, fixed doses are rarely used in clinical practice as the flexible dosing of the medicine is more relevant to patient care [22, 23]. Contrary to fixed dose studies, flexible dose studies suggest that the optimal dose of the OMT medicine should be tailor-made and differ between patients, to account for differences in severity of addiction, chronicity, main substance of dependence, method of administration, potency of main opioid used, tolerance acquired, and idiosyncratic issues.

While numerous reports indicate that the co-abuse of opioids and BZD is ubiquitous around the world, the reasons for the co-abuse of these medications are not entirely clear [24]. Besides the desire to become intoxicated [25], craving and withdrawal symptoms associated with the abused drug are certainly essential causes of co-abuse. Furthermore, a significant relationship has been found between inadequate doses of the OMT medicines experienced by the OMT patients and their BZD-positive urine samples [26]. Although the LC-TOFMS method used in this finding [26] was capable of detecting N-PPM and NPS, those compounds were not found at that time.

The emergence of new abused substances on the market markedly affects the therapy circumstances of OMT patients [1, 7]. However, studies focusing on polydrug abuse taking advance of definitive laboratory methods are few. In this study, our first objective is to evaluate the polydrug abuse among OMT patients, including NPS and N-PPM in addition to traditional illicit drugs, by means of comprehensive LC-TOFMS urine screening. Our second objective is to assess whether polydrug abuse is related to the adequacy of the dose (adequate or inadequate) of the OMT medicine as experienced by the patients.

## Methods

### Procedure

This is a retrospective register-based study for which the data was collected from medical files between November 2015 and January 2016 at the outpatient clinic for opioid-dependent patients of the Helsinki University Central Hospital (HUCH). The clinic is a specialized tertiary addiction psychiatry clinic for opioid-dependent patients who have different psychiatric and somatic comorbidities. Before starting the OMT, all opioid-dependent patients had at least one unsuccesful trial for withdrawal from opioids at some other clinic than at the outpatient clinic for opioid-dependent patients of HUCH. The occurrence of comorbid psychiatric disorders and substance dependencies of the patients (the DSM-IV, Diagnostic and Statistical Manual of Mental Disorders, Fourth Edition) [27] are shown in Table 1. Since our previous study [26], the routine medical files of the clinic have included self-reports regarding substance abuse, withdrawal ratings, cravings, and for experienced adequacy of the dose of the OMT medicine. The rating for dose adequacy can be too low (the dose if insufficient), adequate (the dose if sufficient), too high, or unsure, according to the patient’s opinion. In routine clinical practice, the trough blood concentration of (*R,S*)-methadone was analyzed, but no quantification of buprenorphine in blood was carried out. The routine medical files also included both conventional urine immunoassays for abused drugs taken from one to four times per month and urine LC-TOFMS analysis taken usually once per month. A two-month period for this retrospective study was considered sufficient to obtain one urine LC-TOFMS analysis result from each of the OMT patients. The inclusion criteria for patients were as follows: the dose adequacy rating was either inadequate (Group IA) or adequate (Group A); the urine sample for LC-TOFMS analysis was collected maximally 3 days after the above-mentioned clinical assessments; and the blood sample for methadone quantification was collected the next day after the clinical evaluations.

**Table 1:** Psychiatric comorbid disorders and substance dependences at the start of opioid maintenance treatment^a^

	Total (*n* = 60)	Inadequate dose, *n* = 39 (65.0%)	Adequate dose, *n* = 21 (35.0%)
Number of groups of comorbid psychiatric disorders	1.3 ± 0.70 (0–3)	1.28 ± 0.69 (0–3)	1.33 ± 0.73 (0–3)
Schizophrenia and other psychotic disorder	30 (50.0%)	19 (31.7%)	11 (18.3%)
Mood disorder	9 (15.0%)	5 (8.3%)	4 (6.7%)
Anxiety disorder	10 (16.7%)	6 (10.0%)	4 (6.7%)
Disorders usually first diagnosed in Infancy, Childhood, or Adolescence^b^	2 (3.3%)	2 (3.3%)	0 (0%)
Eating disorder^c^	1 (1.7%)	1 (1.7%)	0 (0%)
Personality disorder	26 (43.3%)	17 (28.3%)	9 (15.0%)
Number of groups of non-opioid drug dependences	1.40 ± 0.89 (0–3)	1.41 ± 0.85 (0–3)	1.38 ± 0.97 (0–3)
Non-opioid drug dependency	51 (85.0%)	35 (58.3%)	16 (26.7%)
Sedative-, hypnotic-, or anxiolytic-related disorder^d^	38 (63.3%)	25 (41.7%)	13 (21.7%)
Amphetamine	19 (31.7%)	13 (21.7%)	6 (10.0%)
Cocaine	0 (0%)	0 (0%)	0 (0%)
Alcohol	12 (20.0%)	6 (10.0%)	6 (10.0%)
Cannabis	6 (10%)	2 (3.3%)	4 (6.7%)
Medications	0 (0%)	0 (0%)	0 (0%)
Polysubstance-related disorder^e^	3 (5.0%)	3 (5.0%)	0 (0%)

^a^All diagnoses are made according to the DSM-IV. There were no statistically significant differences between the two groups of patients

^b^One patient with Mild Mental Retardation and one patient with Attention-Deficit/Hyperactivity Disorder

^c^One patient with Bulimia Nervosa

^d^Two patients with pregabalin dependence

^e^Polysubstance-related disorder was rated as three

Sixty of the 85 different patients (71%) fulfilled the above-mentioned criteria.

The institutional Review Board of the Department of Psychiatry, HUCH, approved the study protocol, which was conducted in accordance with the ethical guidelines set forth by the Declaration of Helsinki.

### Clinical assessments

The opioid withdrawal symptoms as experienced by the patient were based on the short, 10-item withdrawal scale (SOWS) [28] which rates the severity of each withdrawal symptom on a four-point scale, including zero, one, two and three: where zero equals ‘not at all’ and three equals ‘severe’ (range 0–3). The objective rating of withdrawal symptoms was based on the 13-item OOWS scale [29] which indicates each withdrawal symptom on a two-point scale (zero and one): where zero equals ‘absence of any symptom’, and one equals ‘presence of a symptom’. The craving for opioids during the preceding 24 h was based on the visual analog scale (VAS). This study used a single-item VAS rating for the evaluation of craving for opioids: where zero equals ‘none’ and 10 equals ‘very much’. The rating of the withdrawal symptoms and the rating of the craving for opioids were included as potential indicators of the dose adequacy rating.

Thirty-five patients in Group IA, and 17 patients in Group A, carried hepatitis-C but none of the patients had received medication for the disease. One Group A patient had HIV infection which was medicated with Triumeq (dolutegravir/abacavir/lamivudine). Neither the patients with hepatitis-C, nor those with HIV infection, were medicated with opioids or gabapentinoids. None of the patients had any advanced kidney disease which would have indicated medications.

### Laboratory analyses

All patient urine samples were collected under supervision (a one-way mirror), and laboratory analyses were carried out blinded to the patients’ clinical condition. Urine drug screening was performed using the LC-TOFMS method [14]. The in-house LC-TOFMS database included approximately 700 compound entries, including traditional illicit drugs, commonly abused N-PPM, and various classes of NPS, such as non-medical benzodiazepines, synthetic cannabinoids, cathinones, opioids, phenethylamines, piperazines, and tryptamines. A reference standard was available for 400 compounds while the remaining entries were rare NPS and their known or predicted metabolites, for which a reference standard was unavailable. Typical reporting limits in urine for the substances studied were as follows: 50 or 100 ng/mL for BZD, 100 ng/mL for amphetamines, 100 ng/mL for the cocaine metabolite benzoylecgonine, 10 ng/mL for cannabis, 20 ng/mL for NPS, 1 ng/mL for buprenorphine, and 50 ng/mL for other opioids. Among the following psychotropic medicines, prescribed by the attending physicians for the study patients at the out-patient clinic for opioid-dependent patients of HUCH, the LC-TOFMS database included quetiapine but did not include agomelatine, aripiprazole, clozapine, doxepin, escitalopram, fluoxetine, lamotrigine, lithium, melatonin, mirtazapine, olanzapine, paliperidone, risperidone, sertindole, valproate, venlafaxine, or ziprasidone. Gamma-hydroxy butyrate was not analysed due to its very short elimination half-life.

Trough methadone concentrations in serum were determined by gas chromatography – mass spectrometry (GC-MS) in selected ion monitoring mode following liquid-liquid extraction. The limit of quantification (LOQ) was 100 ng/mL, and the expanded uncertainty of measurement was 11%.

### Evaluation of polydrug abuse

Evaluation of the polydrug abuse was carried out according to the DSM-IV. DSM-IV groups the abused substances as follows: 1. alcohol; 2. amphetamine or similarly acting sympathomimetics; 3. caffeine; 4. cannabis; 5. cocaine; 6. hallucinogens; 7. inhalants; 8. nicotine; 9. opioids; 10. phencyclidine (PCP) or similarly acting arylcyclohexyl-amines; 11. sedatives, hypnotics, and anxiolytics; 12. polysubstance abuse; and 13. other abused substances. The positive urine findings by LC-TOFMS were divided following the DSM-IV into six groups of abused substances: 1. amphetamines; 2. BZD corresponding to the sedatives, hypnotics, and anxiolytics group; 3. cannabis; 4. opioids; and 5. NPS and N-PPM belonging to the other abused substance group. The amphetamines group included the common abused substances amphetamine, methamphetamine, 3, 4- methylenedioxymethamphetamine (MDMA, ecstasy)**,** and methylphenidate. The cannabis group consisted of 11-nor-9-carboxy-tetrahydrocannabinol (THC-COOH), which is the main metabolite of tetrahydrocannabinol (THC). The NPS group substances met the criteria for NPS valid during the study [30] as follows: NPS are substances of abuse, either in a pure form or a preparation, that are not controlled by the 1961 Single Convention on Narcotic Drugs or the 1971 Convention on Psychotropic Substances, but which may pose a public health threat. The term ‘new’ does not necessarily refer to new inventions–several NPS were first synthesized 40 years ago–but to substances that have recently become available on the market. After the study, in April 2016, UNDOC placed alpha-pyrrolidinovalerophenone (alpha-PVP) under international control [30].

The N-PPM group included those substances which are not controlled under the Finnish narcotics legislation and were not prescribed by physicians of HUCH, according to the updated electronic database used by both the psychiatric and somatic units of the hospital. The positive urine samples for prescribed medicines were excluded from the study, comprising the 14 urine samples positive for quetiapine and two urine samples positive for oxazepam. All those medicines were prescribed by the physicians of the outpatient clinic for opioid-dependent patients. The use of N-PPM was against the written patient contract given by all the study patients. According to that contract, patients were not denied the use of any medication that is indicated by their somatic disease, but they had to inform the attending physician of the out-patient clinic for opioid-dependent patients of HUCH. We also studied the available self-reports of the patients concerning their abuse of the N-PPM group medicines. The available data showed that pregabalin was abused in five cases, gabapentin in one case, and bupropion in one case. We were unaware as to whether the medicines were prescribed by a physician who was not working at HUCH, whether they were diverted, or whether they were illegally imported to Finland. Any substance included in the above groups had to be backed by some published evidence concerning its abuse potential. We also included the unambiguous metabolites of the substances. A positive result for any abused substance was always based on the positive urine finding by LC-TOFMS and not on self-reports by the patients.

### OMT medicines

Methadone patients received Methadone Martindale Pharma 2 mg/mL oral solution (Martindale Pharmaceuticals Limited, Romford, UK). Buprenorphine patients received a Suboxone buprenorphine-naloxone sublingual tablet containing buprenorphine and naloxone at a ratio of 4:1 (Reckitt Benckiser Healthcare Ltd., Slough, UK).

### Psychosocial treatment

A case management approach providing medical services, community services, and counselling was offered to all patients by the staff of the clinic. As an integrated service, the comorbid psychiatric disorders were treated by the two psychiatrists of the clinic.

### Statistical analysis

Data was reported as the mean ± SD, minimum-maximum values. Two group comparisons were performed with Fisher’s Exact Test for nominal variables and with the T-test for Equality of Means (equal variances not assumed) for continuous variables. The correlations were calculated with the Pearson rank correlation. The tests were two-tailed. The significance level was set at alpha = 0.05. The analyses were performed with SPSS version 22.

## Results

Group IA included 39 (65.0%) patients, and Group A included 21 (35.0%). Neither the gender (F 23.3%) nor the age of the patients (35.4 ± 7.8, 20–56 years) were different between Group IA and A patients (13.3% vs.10.0% and 35.3 ± 7.8, 20–56 years vs. 35.8 ± 8.0, 22–54 years, respectively). At baseline, 56 (93.3%) of the patients had a comorbid psychiatric disorder and 51 (85.0%) of the patients had a non-opioid drug dependency (Table 1). There were no differences in the number of the psychiatric comorbid disorders nor non-opioid drug dependences between Group IA and A patients.

The data of the OMT medicines is shown in Table 2. The number of methadone and buprenorphine/naloxone patients was different between Groups IA and A (*p* = 0.002). No dosage differences for the OMT medicines were evident between Groups IA and A. Similarly, no difference in blood concentration of methadone was detected between Groups IA and A. The blood concentration of methadone was not available for the HIV infected patient medicated with the combination of dolutegravir/abacavir/lamivudine. This study did not find any patients whose opioid or gabapentinoid medications would have affected the methadone blood concentrations.

**Table 2:** The maintenance treatment medicines

	Total sample (*n* = 60)	Inadequate dose (*n* = 39)	Adequate dose (*n* = 21)	*p*
Methadone / Buprenorphine – naloxone sublingual tablet	52 (86.7%) / 8 (13.3%)	38 (63.3%) /1 (1.7%)	14 (23.3%) /7 (11.7%)	0.002
Dose of methadone (mg)	64.81 ± 13.6 (20–90)	65.0 ± 11.4 (20–90)	64.3 ± 18.6 (24–90)	NS
Blood concentration of methadone (mg/L) (*n* = 45)^a^	0.20 ± 0.1 (0.09–0.47)	0.19 ± 0.07 (0.09–0.36) *N* = 35	0.24 ± 0.10 (0.12–0.47), *N* = 10	NS
Dose of buprenorphine (mg)	14.8 ± 5.01 (6–20)	16.0	14.6 ± 5.4 (6–20)	NS

^a^In case the methadone blood concentration was <0.10 mg/L, the value was rated as 0.09 mg/L

The duration of the OMT was shorter in Group IA than in Group A (74.9 ± 75.7, 8–264 weeks vs. 236.3 ± 343.6, 16–512 weeks, *p* = 0.046). There was no correlation between the duration of OMT and polydrug abuse regarding the number of groups of abused substances (*r* = −0.070).

Table 3 demonstrates that the scores for SOWS and for craving were higher in Group IA patients than in Group A patients (*p* = 0.001 and *p* < 0.001, respectively) but there was no difference in the OWS scores.

**Table 3:** Withdrawal symptoms and craving for opioids

	Total sample (*n* = 60)	Inadequate dose (*n* = 39)	Adequate dose (*n* = 21)	*p*
SOWS^a^	8.8 ± 5.9 (0–24)	10.4 ± 6.1 (0–24)	5.8 ± 4.1 (0–18)	0.001
OOWS^b^	0.8 ± 1.4 (0–5)	1.0 ± 1.6 (0–5)	0.5 ± 0.9 (0–3)	NS
Craving for opioids^c^	3.8 ± 2.7 (0–10)	4.7 ± 2.7 (1–10)	2.1 ± 1.6 (0–5)	< 0.001

^a^The short opiate withdrawal scale, range 0–30

^b^The objective rating on withdrawal symptoms, range 0–13

^c^The craving for opioids during the preceding 24 h on visual analog scale, range 0–10

Table 4 shows that Group IA patients had significantly more positive urine samples for abused substances than Group A patients (51.7% vs.13.3%, *p* = 0.002). The number of groups of abused substances was greater in Group IA patients than in Group A patients (2.0 vs 0.9, *p* = 0.005). Both the BZD and amphetamine-containing urine samples were more often positive in Group IA patients than in Group A patients (40.0% vs. 8.3%, *p* = 0.007 and 35.0% vs. 6.7%, *p* = 0.013, respectively). There were 17 (28.3%) urine samples which were positive for both BZD and amphetamines. Fifteen of these samples were provided by Group IA patients and two were provided by Group A patients (*p* = 0.019). The three buprenorphine-positive urine samples were provided by the methadone patients. Group A patients provided no methamphetamine-, fentanyl-, NPS-, gabapentin-, quetiapine- or methylphenidate-positive urine samples but these were all detected in Group IA patients’ samples.

**Table 4:** Polydrug abuse based on LC-TOFMS urine analyses^a^

	Total sample (*n* = 60)	Inadequate dose (*n* = 39)	Adequate dose (*n* = 21)	*p*
Positive urine samples	39 (65.0%)	31 (51.7%)	8 (13.3%)	0.002
Number of groups of abused substances	1.6 ± 1.5 (0–4)	2.0 ± 1.5 (0–4)	0.9 ± 1.4 (0–4)	0.005
BZD-positive	29 (48.3%)	24 (40.0%)	5 (8.3%)	0.007
Oxazepam	23 (38.3%)	19 (31.7%)	4 (6.7%)	0.029
Temazepam	21 (35.0%)	17 (28.3%)	4 (6.7%)	NS
Desmethyldiazepam	17 (28.3%)	15 (25.0%)	2 (3.3%)	0.019
Alprazolam	10 (16.7%)	9 (15.0%)	1 (1.7%)	NS
Clonazepam	7 (11.7%)	6 (10.0%)	1 (1.7%)	NS
Midazolam	3 (5.0%)	3 (5.0%)	0 (0%)	NS
Nitrazepam	2 (3.3%)	1 (1.7%)	1 (1.7%)	NS
Bromazepam	1 (1.7%)	1 (1.7%)	0 (0%)	NS
Demoxepam	1 (1.7%)	1 (1.7%)	0 (0%)	NS
Lorazepam	1 (1.7%)	1 (1.7%)	0 (0%)	NS
Amphetamine-positive	25 (41.7%)	21 (35.0%)	4 (6.7%)	0.013
Amphetamine	25 (41.7%)	21 (35.0%)	4 (6.7%)	0.013
Methamphetamine	2 (3.3%)	2 (3.3%)	0 (0%)	NS
Methylphenidate	1 (1.7%)	1 (1.7%)	0 (0%)	NS
Cannabis-positive	18 (30.0%)	13 (21.7%)	5 (8.3%)	NS
Opioid-positive	4 (6.7%)	3 (5.0%)	1 (1.7%)	NS
Buprenorphine	3 (5.0%)	2 (3.3%)	1 (1.7%)	NS
Norbuprenorphine	1 (1.7%)	1 (1.7%)	0 (0%)	NS
Fentanyl	1 (1.7%)	1 (1.7%)	0 (0%)	NS
NPS-positive	5 (8.3%)	5 (8.3%)	0 (0%)	NS
Alpha-PVP^b^	2 (3.3%)	2 (3.3%)	0 (0%)	NS
MPA^c^	1 (1.7%)	1 (1.7%)	0 (0%)	NS
PV8^d^	3 (5.0%)	3 (5.0%)	0 (0%)	NS
N-PPM-positive^e^	15 (25.0%)	12 (20.0%)	3 (5.0%)	NS
Pregabalin	10 (16.7%)	8 (13.3%)	2 (3.3%)	NS
Gabapentin	2 (3.3%)	2 (3.3%)	0 (0%)	NS
Quetiapine	3 (5.0%)	3 (5.0%)	0 (0%)	NS
Bupropion	2 (3.3%)	1 (1.7%)	1 (1.7%)	NS

^a^Liquid chromatography coupled to high-resolution time-of-flight mass spectrometry (LC-TOFMS)

^b^Alpha-pyrrolidinovalerophenone

^c^Methiopropamine

^d^a-PHPP or alpha-pyrrolidinoheptiophenone

^e^Non-prescribed psychotropic medicines

Table 5 shows the data for the patients receiving daily methadone doses less than 60 mg and for those receiving daily buprenorphine doses less than 16 mg. Among these patients, all urine samples positive for abused substances were privided by Group IA patients.

**Table 5:** Polydrug abuse of the patients with low doses of the maintenance treatment medicine

Medicine	Dose (mg)	Adequacy of the dose of the medicine	Blood concentration (mg/L)	Abused substances based on LC-TOFMS method^a^
Methadone	20	Too low dose	< 0.10	Alprazolam
Methadone	24	Adequate	Not available	No substances
Methadone	30	Adequate	0.17	No substances
Methadone	50	Too low dose	< 0.10	Oxazepam, temazepam, amphetamine, bupropion
Buprenorphine	6	Adequate	Not available	No substances
Buprenorphine	8	Adequate	Not available	No substances

^a^Liquid chromatography coupled to high-resolution time-of-flight mass spectrometry (LC-TOFMS)

## Discussion

### Polydrug abuse

This study found widespread polydrug abuse among OMT patients as 65% of the urine samples tested positive for abused drugs via LC-TOFMS analysis (Table 4). Abused substances were found in all six studied groups of drugs, in order of decreasing occurrence: BZD (48%), amphetamines (42%), cannabis (30%), N-PPM (25%), NPS 8%, and opioids (7%). Polydrug abuse, in terms of the number of groups of abused substances, was significantly more common among Group IA patients than among Group A patients. Group IA patients had more positive urine samples for abused drugs (52% vs.13%), more BZD-positive urine samples (40% vs. 8%) and more amphetamine-positive urine samples (35% vs. 7%) than Group A patients. All the NPS-positive samples, containing alpha-PVP, MPA (methiopropamine), and PV8 (alpha-PHPP, alpha-pyrrolidinoheptiophenone), were provided by Group IA patients. Most of the N-PPM-positive urine samples, containing pregabalin, gabapentin, and quetiapine, were from Group IA patients. In addition, Group IA patients experienced more subjective withdrawal symptoms and cravings for illicit opioids as potential indicators for the dose-adequate rating. Neither the doses of the OMT medicines, nor the methadone blood concentrations, were different between the two groups of patients.

The 40% occurrence of BZD-positive urine samples among patients with inadequate doses is almost the same (38%) as in our previous study among a similar group of OMT patients [26]. At baseline, 42% of Group IA patients of the present study had a sedative-, hypnotic, or anxiolytic related disorder, whereas 90% of the patients in our previous study [26] had either BZD abuse or dependence at the baseline. These findings do not exclude the possibility that methadone treatment may trigger the onset or worsening of BZD abuse as pointed out by Chen et al. [10]. Group IA patients also demonstrated significant co-abuse of BZD and amphetamines. Regarding individual BZD drugs, oxazepam (38%), temazepam (35%), and desmethyldiazepam (28%) were those most commonly found. The number of urine samples positive for abused temazepam and oxazepam would have been lower if these compounds had been considered the metabolites of diazepam. Diazepam has a higher abuse liability than oxazepam [24, 31], and consequently it is possible that many of the oxazepam-and temazepam-positive urine samples of this study reflect the abuse of diazepam.

Amphetamine-positive urine samples were found significantly more often among Group IA patients (35%) than Group A patients (7%). The importance of prescribing appropriate methadone dosages to indirectly reduce cocaine use has been described in the study by Baumeister et al. [32]. Their consideration is in line with the present study concerning amphetamine. Group IA patients had two methamphetamine-positive urine samples. An earlier Finnish study found 21% methamphetamine-positive urine samples among OMT patients with irregular attendance to a harm reduction unit, but no methamphetamine-positive urine samples among OMT patients with regular attendance to drug treatment at a rehabilitation clinic [16]. Regular visits to the clinic are inevitably related to more adequate doses of the OMT medicine than irregular visits. Cocaine is seen in the Finnish drug market [4], and also among OMT patients [15, 16], infrequently. Those reports are in line with the negative urine samples of this study regarding the cocaine metabolite benzoylecgonine.

Most of the gabapentinoid (gabapentin and pregabalin)-positive urine samples (17%) were found among Group IA patients. Up to 32% pregabalin-positive urine samples have been found among Finnish OMT patients who irregularly attend drug treatment at a harm reduction unit [16]. Both gabapentin and pregabalin are approved for the management of neuropathic pain [33] and it is possible that the abuse of those drugs is related at least partly to control opioid withdrawal symptoms such as pain, unrest, and sleeplessness, but also due to the psychotropic effects of the gabapentinoids [17]. No gabapentinoids prescribed for somatic diseases were found in this study.

Extremely common misuse of quetiapine among clients of a methadone maintenance program has been reported by McLarnon et al. [34]. All the three quetiapine-positive urine samples of the present study were provided by Group IA patients. Quetiapine has been reported to increase plasma concentrations of (R)-methadone [35]. No high-methadone blood concentrations were found in the two methadone patients from our study, who abused quetiapine. These patients demonstrated 0.17 mg/L and 0.29 mg/L methadone corresponding to the 80 mg and 74 mg daily doses, respectively.

The present study found five (8%) NPS-positive urine samples which all were given by Group IA patients. The synthetic cathinones PV8 and alpha-PVP, and the thiophene ring-based structural analog of methamphetamine MPA, were found in three, two, and one sample, respectively. PV8 appeared on the illicit drug market in 2013 as a candidate to replace MDPV [36]. No MDPV was detected in this study, in contrast to our previous studies among OMT patients [15, 26]. To our knowledge, this study represents the first report on the abuse of PV8 among OMT patients.

Cannabis-positive urine samples (30%) were found both in Group IA (22%) and in Group A (8%). The present study detected only THC-COOH and no synthetic cannabinoids, similar to previous Finnish studies [15, 16], although the LC-TOFMS method used in those studies is able to detect many synthetic cannabinoids. The absence of synthetic cannabinoids is likely due to the increased incidence of home growing of cannabis in Finland [4].

Opioids (7%) were the most seldom-found group of abused substances. Only 2% of urine samples were opioid-positive among Group A patients. Similarly, Finnish OMT patients with regular attendance for drug treatment had 7% opioid-positive urine samples in contrast to 21% opioid-positive urine samples if the attendance of the OMT patients was irregular [16]. Thus, the primary task of OMT, to prevent abuse of opioids, is well realized when the dose of the OMT medicine is adequate and the OMT patients regularly attend their clinic. However, the three buprenorphine positive urine samples from patients in methadone treatment are alarming because of potential severe withdrawal symptoms caused by the interaction of those substances.

### Doses of the OMT medicines and polydrug abuse

No differences were observed in the mean daily doses of methadone or buprenorphine/naloxone between Group IA and Group A patients, i.e., 65 mg vs. 64 mg and 16 mg vs. 15 mg, respectively. The 65 mg mean daily dose of methadone in this study is at the recommended level (60 – 100 mg) according to reported fixed high-dose studies [19–21], but the mean 15 mg daily dose of buprenorphine is somewhat lower than the minimum effective mean daily dose of buprenorphine (16 mg) in terms of suppression of abuse of opioids among heroin-dependent patients [2]. Nonetheless, smaller doses of OMT medicines have also been effective in reducing substance abuse. In a flexible dose study, Soyka et al. [23] reported that the mean daily doses of 44–50 mg methadone and 9–12 mg of buprenorphine were related to a significant decrease in substance use. Furthermore, concomitant drug use for all illicit substances decreased with either an 8 mg or 10 mg daily dose of buprenorphine after 12 months in a non-interventional study performed under real-life conditions [12]. The real life conditions of the study by Apelt et al. [12] were similar to those of the present study, where even 24 mg and 30 mg daily doses of methadone, and 6 mg and 8 mg daily doses of buprenorphine, were adequate and urine samples were negative for abused substances (Table 5).

### Methadone blood concentration and polydrug abuse

No difference was evident in mean methadone trough blood-concentration (0.20 mg/L) between Group IA (0.19 mg/L) and Group A (0.24 mg/L) patients. Two Group IA methadone patients receiving 20 mg and 50 mg daily doses had less than 0.10 mg/L methadone blood concentrations (Table 5). Group IA also included two other methadone patients (60 mg and 76 mg daily doses) whose methadone blood-concentrations were less than 0.10 mg/L. We found that a methadone concentration less than 0.10 mg/L is related to polydrug abuse, thus higher methadone blood concentrations are needed. The mean methadone concentration of this study was lower than the 400 ng/mL reported by D’Aunno et al. [21]. It is possible that somewhat higher methadone blood-concentrations would have been combined with less polydrug abuse. However, the blood concentration of methadone has large interindividual variation for a given dosage due to the interindividual variability of CYP enzymes and interactions between methadone and several medications [37]. In addition, both cannabis and BZD can affect methadone blood concentrations [38]. Therefore, it is interesting that there is no evidence of a greater variability in the blood concentrations of methadone among the patients with polydrug abuse and different psychiatric comorbidities (Table 2).

### Strengths and limitations

This study carries several limitations. The cross-sectional design limits making causal and temporal conclusions between the dose adequacy and polydrug abuse among OMT patients. In addition, the substance groups per definition in some way may overlap and may share similar features. Furthermore, the number of patients was relatively small, giving rise to possible statistical type I and II errors.

Regarding the OMT medicines, Group IA patients were more often on methadone than Group A patients. The patients were not randomized to methadone and buprenorphine/naloxone medication at the start of the OMT, and it is possible that patients with more severe opioid dependence were started with methadone. In addition, the total number of buprenorphine/naloxone patients was low.

The occurrence of individual abused substances in urine samples cannot be generalized because substance abuse in different countries is highly variable and dependent on both the area, the period of evaluation, and the target population. The LC-TOFMS method was limited by the content of the database used. Although full high-resolution MS data were acquired, the data analysis protocol mined only for the masses included in the database. If any of the patients were using a drug that is not targeted by the method, it is highly likely that a false negative result would have been obtained. The LC-TOFMS database of this study did not include olanzapine and venlafaxine which both possess abuse liability [39]. However, this study shows that OMT patients abuse a broad range of illicit and licit substances and it is unlikely that this is only a Finnish feature among OMT patients. Concerning the safety of the OMT, it is important to give the OMT patients the correct information regarding the abused substances because the patients are seldom aware of the actual substances they are abusing [15]. The uncertainty of the patients concerning their abused substances may partly be related to the psychiatric comorbidities and to impaired memory among methadone or buprenorphine patients using BZD [40]. Alcohol consumption was not controlled in this study. A third of patients receiving OMT have been found to have increased alcohol consumption and alcohol use disorders [41]. Furthermore, a recent study by Preston et al. [42] reports that drinking was associated with heroin and cocaine craving and actual use among patients in methadone maintenance treatment. Thus, the lack of data concerning alcohol consumption is a clear limitation of this study.

Group IA patients demonstrated moderately high mean scores for opioid craving (4.7), whereas Group A patients had quite low mean scores for craving (2.1) on the VAS-scale. Craving may have different roles among those using various substances and craving contains both automatic and cognition-controlled processes [43, 44]. The VAS-scale may have measured global craving for drugs, although the patients in the present study were advised to focus only on craving due to illegal opioids as the patients had much polydrug abuse and many psychiatric comorbidities. Besides the uncertainty of rating for craving focusing on only opioids, the rating of subjective withdrawal symptoms may have been nonspecific to some extent, because BZD abuse may also exacerbate opioid-specific withdrawal symptoms [45].

This study failed to consider the actual distress/symptoms related to the comorbid psychiatric disorders or the licit psychotropic medicines prescribed by the attending physicians. Although the occurrence of the drug dependences and the comorbid psychiatric disorders at baseline were not different between Group IA and Group A patients, the actual psychiatric syndrome and the licit psychotropic medicines might have been different between the two groups and consequently might have affected polydrug abuse differently. This study cannot exclude the possibility that some of the abused substances were actually targeted to treat some of the drug dependences or psychiatric disorders. The more than 90% occurrence of comorbid psychiatric disorders of this study by itself is similar to the study of Brooner et al. [46]. Interestingly, Brooner et al. [46] found that in methadone maintenance treatment, reductions in psychiatric distress of the patients were unrelated to substance use outcomes.

It is possible that there are important additional factors that are related to the dose adequacy rating and can provide more information about polydrug abuse in OMT. The scores of stability of sense of coherence were similar at baseline and after 1 year among patients in methadone maintenance treatment, but were lower among patients who still abused any drugs compared with patients who did not [47]. Thus, the relationship between dose adequacy rating and the sense of coherence rating would be an interesting topic in future studies on polydrug abuse among OMT patients.

## Conclusions

This study detected widespread polydrug abuse among OMT patients based on comprehensive LC-TOFMS urine screening. Polydrug abuse was more common if the dose of the OMT medicine was too low and thus inadequate as experienced by the patients answering a simple questionnaire. Inadequate doses of the OMT medicines were associated with higher subjective withdrawal scores and craving for opioids which are potential indicators for the dose adequacy rating. Additional causes of polydrug abuse concerning different groups of abused substances clearly merits future studies among OMT patients.

## Abbreviations

Alpha-PVP: alpha-pyrrolidinovalerophenone
BZD: Benzodiazepine
CYP: Cytochrome P450 enzyme
DSM-IV: Diagnostic and Statistical Manual of Mental Disorders, Fourth Edition
EMCDDA: European Monitoring Centre for Drugs and Drug Addiction
GC-MS: Gas chromatography – mass spectrometry
Group A: Opioid-dependent patients in opioid maintenance treatment with adequate doses of the opioid maintenance treatment medicine as experienced by the patients
Group IA: Opioid-dependent patients in opioid maintenance treatment with inadequate doses of the opioid maintenance treatment medicine as experienced by the patients
LC-TOFMS: Liquid chromatography - high-resolution time-of-flight mass spectrometry
LOQ: Limit of quantification
MDMA: 3, 4- methylenedioxymethamphetamine (ecstasy)
MPA: Methiopropamine
N-PPM: Non - prescribed psychotropic medicines
NPS: New psychoactive substances
OMT: Opioid maintenance treatment
OOWS scale: Objective Opiate Withdrawal Scale
PV8: a-PHPP, alpha-pyrrolidinoheptiophenone
SD: Standard deviation
SOWS scale: Short opiate withdrawal scale
THC: Tetrahydrocannabinol
THC-COOH: 11-nor-9-carboxy-tetrahydrocannabinol
UNDOC: United Nations Office of Drugs and Crime
VAS: Visual analog scale
